# Multi-dimensional excited-state energy modulation of NIR-II nanoaggregates for phototheranostics

**DOI:** 10.1093/nsr/nwaf407

**Published:** 2025-09-23

**Authors:** Wei-Hong Zhu

**Affiliations:** Shanghai Key Laboratory of Functional Materials Chemistry, Key Laboratory for Advanced Materials and Institute of Fine Chemicals, Joint International Research Laboratory of Precision Chemistry and Molecular Engineering, Feringa Nobel Prize Scientist Joint Research Center, Frontiers Science Center for Materiobiology and Dynamic Chemistry, School of Chemistry and Molecular Engineering, East China University of Science and Technology, China

Phototheranostics, which enable simultaneous real-time diagnostic imaging and *in situ* therapeutic intervention under non-invasive light activation, is rapidly emerging as a cutting-edge field in medical treatment [1]. This integrated approach offers significant benefits such as low systemic toxicity, high precision in diagnostic imaging, effective tumor eradication, and minimal development of treatment resistance. Among current systems, single-component organic small molecules capable of delivering both intense second near-infrared (NIR-II) fluorescence imaging and potent phototherapeutic effects have attracted considerable attention [2]. The NIR-II region is especially advantageous for bio-applications due to its superior tissue penetration, reduced light scattering, minimal autofluorescence, and lower photon absorption [3,4]. However, designing high-performance organic phototheranostic agents remains a considerable obstacle. Key challenges stem from the fundamental constraints of the energy gap law, which dictates that fluorescence emission weakens at longer wavelengths due to increased non-radiative decay rates. Competing radiative and non-radiative pathways further divert photoexcitation energy. As a result, balancing the demands for bright imaging signals and efficient therapeutic outcomes becomes increasingly difficult, severely limiting the performance of phototheranostic agents in deep-tissue applications.

**Figure 1.**
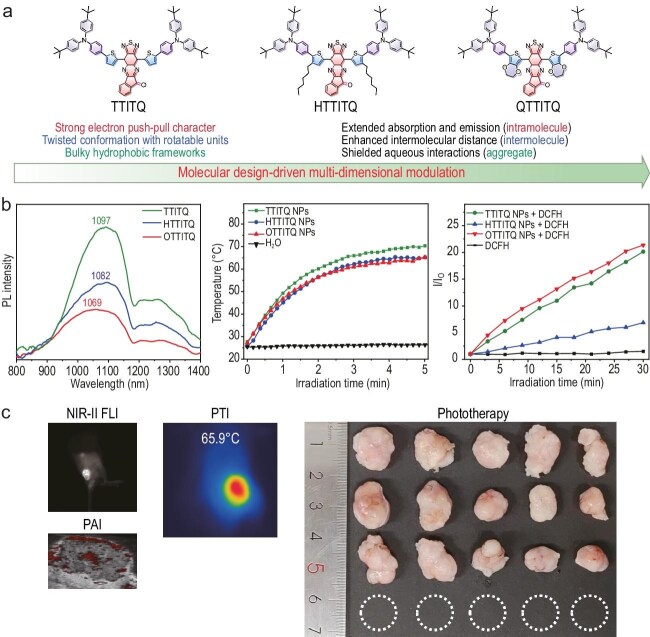
(a) The structural formula and design philosophy of the molecules. (b) Fluorescence emission, photothermal conversion and ROS generation of the molecules. (c) NIR-II fluorescence (FLI)/photoacoustic (PAI)/photothermal (PTI) trimodal imaging-guided synergistic phototherapy.

Recently, in response to the aforementioned challenges, a group led by Prof. Dong Wang [5] at Shenzhen University designed a class of multifunctional organic molecules that exhibit bright NIR-II fluorescence, efficient reactive oxygen species (ROS) generation, and high photothermal conversion performance (Fig. 1). Through delicate molecular engineering, the team

modulated both intrinsic electronic properties and supramolecular aggregation behavior. They introduced a novel strategy termed ‘multi-dimensional excited-state energy modulation’, aimed at enhancing phototheranostic performance through coordinated manipulation at the intramolecular, intermolecular, and aggregate levels. At the intramolecular dimension, the strong electron push-pull character was realized by integrating a 4-(*tert*-butyl)-*N*-(4-(*tert*-butyl)phenyl)-*N*-phenylaniline donor with a 10*H*-indeno[1,2-*b*][1,2,5]thiadiazolo[3,4-*g*]quinoxalin-10-one acceptor, resulting in extended absorption and emission wavelengths. On the intermolecular level, the introduction of twisted molecular conformations facilitated by multiple rotatable triphenylamine units can effectively suppress π-π stacking, thus reducing the aggregation-caused fluorescence quenching. These pronounced molecular motions can further contribute to the enhanced photothermal conversion. Finally, at the aggregate dimension, the incorporation of bulky hydrophobic frameworks is beneficial to shielding the nanoparticle surface from aqueous interactions, thereby preserving fluorescence intensity in aqueous environments. The proposed design concept was rigorously validated through quantum chemical calculations and molecular dynamics simulations.

Guided by this innovative design principle, the optimized molecular system was specifically synthesized and assembled into nanoparticles. These nanoparticles demonstrate an emission maximum at 1084 nm in the NIR-II region, exhibit robust generation of •OH and •O^2−^ via a type I photochemical process, and achieve a photothermal conversion efficiency as high as 35.63%. These results collectively signify a successfully engineered balance between radiative and non-radiative decay processes, which is essential for advanced phototheranostic functionality. In addition to their outstanding optical and phototherapeutic performance, the nanoparticles possess optimal hydrodynamic diameter, excellent aqueous dispersibility, remarkable photostability, and high biocompatibility. Capitalizing on these attributes, the system enables high-precision trimodal (NIR-II fluorescence/photoacoustic/photothermal) imaging-guided synergistic photodynamic and photothermal therapy. As evidenced by experimental results, it facilitates accurate tumor delineation and achieves complete tumor eradication, underscoring its potential for practical theranostic applications.

In summary, the study represents a notable advancement in the development of organic phototheranostic agents exhibiting NIR-II emission, offering a viable pathway to overcome the constraints imposed by the energy gap law and the competitive deactivation of excited-state energy. These findings not only provide valuable insights for creating high-performance multifunctional phototheranostics for disease management, but also illustrate a conceptual transition from molecular design to aggregate-level engineering. This shift opens new avenues for material innovation by exploiting collective properties in the aggregated state that are unattainable in individual molecules, thereby enabling emergent functionalities. The aggregation-regulation strategy presented in this study exhibits broad universality and can be adapted to diverse photosensitizer systems, offering a novel paradigm for achieving high-performance multimodal phototheranostics. This approach holds considerable promise for expanding cancer treatment options and demonstrates strong potential for clinical translation.
